# Assessment of prognostic implication of a panel of oncogenes in bladder cancer and identification of a 3-gene signature associated with recurrence and progression risk in non-muscle-invasive bladder cancer

**DOI:** 10.1038/s41598-020-73642-8

**Published:** 2020-10-06

**Authors:** Constance Le Goux, Sophie Vacher, Anne Schnitzler, Nicolas Barry Delongchamps, Marc Zerbib, Michael Peyromaure, Mathilde Sibony, Yves Allory, Ivan Bieche, Diane Damotte, Geraldine Pignot

**Affiliations:** 1 Pharmacogenomics Unit, Department of Genetic, Curie Institute, Paris, France; 2 Department of Urology, Bicêtre Hospital, Paris Sud University, 78 rue du Général Leclerc, 94270 Le Kremlin-Bicêtre, France; 3 Department of Urology, Cochin Hospital, Paris Descartes University, Paris, France; 4 Department of Pathology, Cochin Hospital, Paris Descartes University, Paris, France; 5 Department of Pathology, Curie Institute, Paris, France; 6 Inserm U1016, Cochin Institute, University Paris Descartes, Paris, France; 7 Cancer, Immune Control, and Escape, INSERM U1138, Cordeliers Research Center, Paris, France; 8 Department of Surgical Oncology, Institut Paoli-Calmettes, Marseille, France

**Keywords:** Urological cancer, Molecular biology, Urology

## Abstract

This study evaluated the prognostic value of a panel of 29 oncogenes derived from the analysis of The Cancer Genome Atlas (TCGA data) or from the recent literature on bladder tumors on a well-characterized series of muscle-invasive bladder cancer (MIBC) and non-MIBC (NMIBC) samples and tried to identify molecular prognostic markers. Mutations of *HRAS*, *FGFR3*, *PIK3CA* and *TERT* were found in 2.9%, 27.2%, 14.9% and 76.7% of tumor samples, respectively. Concerning NMIBC, on multivariate analysis, *RXRA* and *FGFR3* levels were associated with recurrence-free survival (RFS) (*p* = 0.0022 and *p* = 0.0069) and *RXRA* level was associated with progression to muscle-invasive disease (*p* = 0.0068). We identified a 3-gene molecular signature associated with NMIBC prognosis. *FGFR3* overexpression was associated with reduced response to Bacillus Calmette–Guerin treatment (*p* = 0.037). As regards MIBC, on multivariate analysis, *ERCC2* overexpression was associated with RFS (*p* = 0.0011) and *E2F3* and *EGFR* overexpression were associated with overall survival (*p* = 0.014 and *p* = 0.035). RT-PCR findings were confirmed by IHC for FGFR3. Genomic alterations in MIBC revealed in TCGA data also concern NMIBC and seem to be associated with prognosis in terms of recurrence and progression. Correcting these alterations by targeted therapies seems a promising pharmacological approach.

## Introduction

With 430,000 cases diagnosed worldwide, bladder cancer is responsible for 150,000 deaths a year. In France, 11,000 new cases of bladder cancer are diagnosed each year. About 70% of newly diagnosed cases are non-muscle-invasive bladder cancer (NMIBC). These cases have a 60% recurrence rate, and 10% evolve to muscle-invasive tumors. Muscle-invasive bladder cancer (MIBC) represents 30% of cases at diagnosis. Survival greatly differs between NMIBC and MIBC.

Contrary to what was previously thought, focal and recurrent gene amplifications are common in urothelial bladder carcinomas. Recently, genome analysis revealed new genomic alterations that could be used as markers in clinical oncology. Analysis of The Cancer Genome Atlas (TCGA) data revealed 27 amplified regions^1^. Integrated analysis of the various genetic alterations described in TCGA revealed three main deregulated signaling pathways in bladder tumors and potential therapeutic targets. Deregulations affecting the cell cycle were found in 93% of cases, those affecting histone pathway in 89% of cases, those affecting the PI3K/AKT/mammalian target of rapamycin (mTOR) pathway in 72% of cases, and those involved in chromatin remodeling in 64% of cases^1^. However, there are few comparative data in the literature regarding these alterations in NMIBC, probably because of the constraints associated with the sampling procedures inherent in NMIBC.

We aimed to identify genes whose abnormalities are related to prognosis, especially NMIBC prognosis. We studied a panel of 29 oncogenes derived from the analysis of TCGA data or from the recent literature on bladder tumors^1–7^ on a well-characterized series of NMIBC and MIBC samples and tried to identify molecular prognostic markers.

## Results

### DNA mutations

NMIBC and MIBC samples showed mutation in *HRAS* in 4.5% (2/44) and 1.7% (1/59) of cases, respectively; in *FGFR3* in 50.0% (22/44) and 10.2% (6/59) of cases, respectively; in *PIK3CA* in 16.7% (10/60) and 13.5% (9/67) of cases, respectively; and in *TERT* in 79.5% (35/44) and 74.6% (44/59) of cases, respectively (Table 1 and Supplementary Data 1). DNA mutations were not associated with prognosis in terms of recurrence or progression of NMIBC or in terms of RFS or OS of MIBC (data not shown).

**Table 1:** Clinical and pathological characteristics of patients (**a**) 61 patients with non-muscle-invasive bladder cancer (NMIBC). (**b**) 67 patients with muscle-invasive bladder cancer (MIBC).

		No recurrence	Recurrence		Muscle-invasive progression	
	n (%)	n (%)	n (%)	p*	n (%)	p**
**Total population**	61 (100)	19 (31.1)	32 (52.5)		10 (16.4)	
**Age (years)**						
≥ 60	45 (73.8)	13 (68.4)	22 (68.8)	0.98	10 (100.0)	**0.0095**
< 60	16 (26.2)	6 (31.6)	10 (31.2)		0 (0.0)	
**Sex**						
Male	54 (88.5)	17 (89.5)	28 (88.9)	0.81	9 (90.0)	0.70
Female	7 (11.5)	2 (10.5)	4 (11.1)		1 (10.0)	
**Smoking status**						
Non-smoker	27 (44.3)	6 (31.6)	16 (50.0)	0.20	5 (50.0)	0.96
Smoker	34 (55.7)	13 (68.4)	16 (50.0)		5 (50.0)	
**History of NMIBC**						
No	34 (55.7)	16 (84.2)	14 (43.7)	**0.0045**	4 (40.0)	0.45
Yes	27 (44.3)	3 (15.8)	18 (56.3)		6 (60.0)	
**Cis associated**						
No	58 (95.1)	19 (100.0)	31 (96.9)	0.79	8 (80.0)	**0.015**
Yes	3 (4.9)	0 (0.0)	1 (3.1)		2 (20.0)	
**Grade**						
Low	25 (41.0)	10 (52.6)	14 (43.8)	0.48	1 (10.0)	**0.029**
High	36 (59.0)	9 (47.4)	18 (56.2)		9 (90.0)	
**Tumor stage**						
Ta	39 (63.9)	13 (68.4)	23 (71.9)	0.79	3 (30.0)	**0.015**
T1	22 (36.1)	6 (31.6)	9 (28.1)		7 (70.0)	
**HRAS mutation**^£^						
No	42 (95.5)	13 (92.9)	21 (95.5)	0.68	8 (100.0)	0.80
Yes	2 (4.5)	1 (7.1)	1 (4.5)		0 (0.0)	
**FGFR3 mutation**^£^						
No	22 (50.0)	7 (50.0)	11 (50.0)	1.00	4 (50.0)	1.00
Yes	22 (50.0)	7 (50.0)	11 (50.0)		4 (50.0)	
**PIK3CA mutation**^££^						
No	50 (83.3)	13 (72.2)	28 (87.5)	0.18	9 (90.0)	0.53
Yes	10 (16.7)	5 (27.8)	4 (12.5)		1 (10.0)	
**TERT mutation**^£^						
No	9 (20.5)	4 (28.6)	4 (18.2)	0.46	1 (12.5)	0.54
Yes	35 (79.5)	10 (71.4)	18 (81.8)		7 (87.5)	

		Recurrence-free survival		Overall survival		
	n (%)	n (%)^**a**^	p*	n (%)^**b**^	p*	
**Total population**	67 (100)	46 (68.7)		40 (59.7)		
**Age (years)**						
≥ 60	49 (73.1)	39 (79.6)	**0.001**	34 (69.4)	**0.008**	
< 60	18 (26.9)	7 (38.9)		6 (33.3)		
**Sex**						
Male	53 (79.1)	34 (64.2)	0.16	32 (60,4)	0.83	
Female	14 (20.9)	12 (85.7)		8 (57.1)		
**Smoking status**						
Non-smoker	15 (22.4)	13 (86,7)	0.09	12 (80.0)	0.07	
Smoker	52 (77.6)	33 (63.5)		28 (53.8)		
**History of NMIBC**						
No	40 (59.7)	31 (77.5)	0.06	25 (62.5)	0.57	
Yes	27 (40.3)	15 (55.6)		15 (55.6)		
**Tumor stage**						
T2	25 (37.3)	17 (68.0)	0.93	10 (40.0)	**0.01**	
≥ T3	42 (62.7)	29 (69.0)		30 (71.4)		
**N status**						
N-	42 (62.7)	25 (59.5)	0.06	19 (45.2)	**0.002**	
N +	25 (37.3)	21 (84.0)		21 (84.0)		
**HRAS mutation**^£^						
No	58 (98.3)	38 (97.4)	0.48	30 (96.8)	0.43	
Yes	1 (1.7)	1 (2.6)		1 (3.2)		
**FGFR3 mutation**^£^						
No	53 (89.8)	35 (89.7)	0.97	28 (90.3)	0.92	
Yes	6 (10.2)	4 (10.3)		3 (9.7)		
**PIK3CA mutation**^££^						
No	59 (88.1)	41 (89.1)	0.69	35 (87.5)	0.86	
Yes	9 (13.5)	5 (10.9)		5 (12.5)		
**TERT mutation**^£^						
No	15 (25.4)	8 (20.5)	0.23	7 (22.6)	0.60	
Yes	44 (74.6)	31 (79.5)		24 (77.4)		

*Chi-square test (*Recurrence* versus *No recurrence*); **chi-square test (*Muscle-invasive progression* versus *the others*).

^£^Information available for 44 patients; ^££^ Information available for 60 patients.

^a^First recurrence (local or metastatic); ^b^ Death; *chi-square test.

^£^Information available for 59 patients; ^££^ Information available for 67 patients.

Bold values are statistically significant (*p* < 0.05).

### Gene expression

For most genes except *PRKCI*,* FBXW7*,* E2F3*,* SOX4*,* YWHAZ*,* GDI2*,* FRS2*,* ERCC2* and *BCL2L1*, the expression profile differed between MIBC and NMIBC samples (Supplementary Data 2).

In NMIBC samples, 24/29 (82.8%) genes were significantly deregulated as compared with normal bladder tissue (*p* < 0.05) (Supplementary Data 2); 4 genes showed significant differences in expression by stage (pTa versus pT1), 4 by grade (low versus high) and 5 by stage and grade *(TACC3*,* FBXW7*,* SOX4*,* YWHAZ*,* Ki67)* (Supplementary Data 3).

In MIBC samples, 25/29 genes (86.2%) were significantly deregulated as compared with normal bladder tissue (*p* < 0.05) (Supplementary Data 2).

### Association between gene expression and prognosis of NMIBC

On univariate analysis, only the expression of *RXRA*,* FGFR3* and *CCNE1* was associated with RFS (*p* = 0.0017, *p* = 0.043 and *p* = 0.039, respectively) and PFS (*p* = 0.0043, *p* = 0.022 and *p* = 0.022, respectively) (Supplementary Data 4, 5, and 6).

Correlation between *PPARG* and *RXRA* mRNA expressions were strong (*p* < 0.0000001, Spearman’s test); however, *PPARG* was not associated with prognosis.

Multivariate analyses included covariates associated with RFS or PFS showing significance at *p* < 0.05 on univariate analysis (i.e., history of NMIBC; *RXRA*,* FGFR3* and *CCNE1* status for recurrence and stage, grade, Cis and *RXRA*,* FGFR3* and *CCNE1* status for progression to muscle-invasive tumor). On multivariate analysis, history of NMIBC and *RXRA* and *FGFR3* status remained associated with RFS (*p* = 0.0045, *p* = 0.0022 and *p* = 0.0069, respectively) and only *RXRA* status with PFS (*p* = 0.0068) (Table 2).

**Table 2:** Multivariate analysis of prognostic factors involved in recurrence-free survival and progression-free survival in non-muscle-invasive bladder cancer.

Prognostic factor	Recurrence-free survival		
	Adjusted HR	95% CI	p*
History of NMIBC	2.63	[1.35–5.13]	**0.0045**
RXRA overexpression	0.34	[0.17–0.68]	**0.0022**
FGFR3 overexpression	0.39	[0.20–0.77]	**0.0069**
CCNE1 overexpression	1.53	[0.81–2.89]	0.19

Prognostic factor	Progression-free survival		
	Adjusted HR	95% CI	p*
T1 stage	5.93	[0.68–51.34]	0.11
Cis associated	1.52	[0.26–9.08]	0.64
High grade	1.10	[0.06–18.76]	0.95
RXRA overexpression	0.06	[0.01–0.47]	**0.0068**
FGFR3 overexpression	0.12	[0.01–1.00]	0.05
CCNE1 overexpression	5.53	[0.97–31.39]	0.053

*HR* hazard ratio,* 95% CI*  95% confidence interval.

*Cox model.

Bold values are statistically significant (*p* < 0.05).

**Figure 1:**
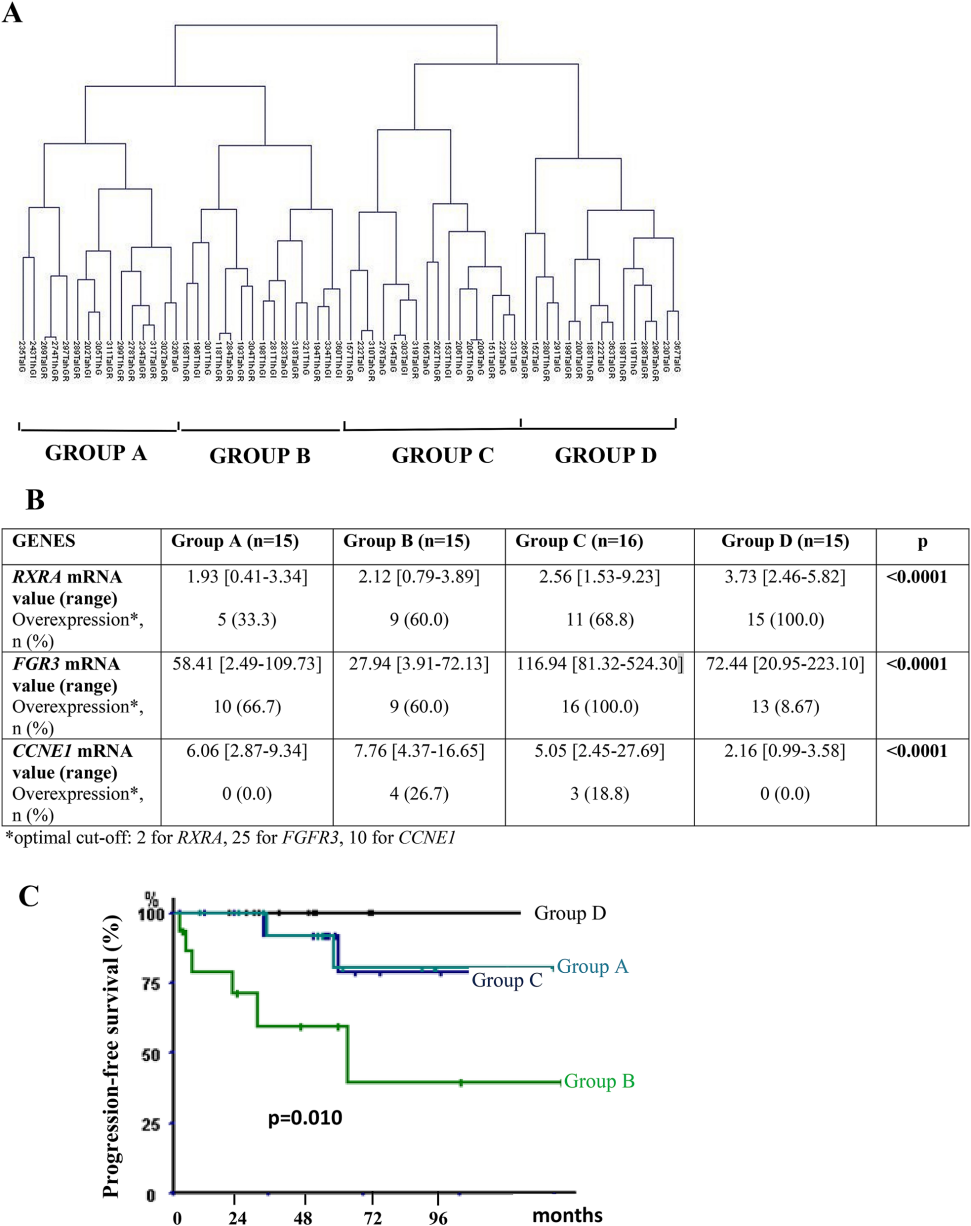
Supervised classification analysis of the 61 NMIBC tumor samples with the 3-gene signature comprising *RXRA*, *FGFR3* and *CCNE1*. (**A**) dendrogram of four tumor groups obtained by hierarchical cluster with the 3-gene signature. (**B**) mRNA median value [range] and overexpression rates of *FGFR3*, *RXRA* and *CCNE1* in the four tumor groups. *p* value calculated by Kruskal Wallis H-test. For each gene, mRNA values ≥ optimal cut-off were considered to represent overexpression and ≤ optimal cut-off, underexpression. (**C**) Kaplan–Meier curves comparing progression-free survival for tumor groups. *p* value calculated by log rank test.

We performed unsupervised hierarchical clustering analyses of 61 NMIBC samples with the 3 genes previously found in NMIBC samples (*RXRA*, *FGFR3*, and *CCNE1*) and found 4 major clusters composed of 15 (Group A), 15 (Group B), 16 (Group C) and 15 (Group D) samples with various mRNA levels of these 3 genes (Fig. 1A,B). The 4 cluster groups significantly differed in terms of PFS (log rank *p* = 0.010): Kaplan–Meier survival curves showed poor outcome associated with group B and better with group D (Fig. 1C). Multivariate analyses including covariates associated with RFS and PFS with significance at *p* < 0.05 on univariate analysis and including the 3-gene signature retained history of NMIBC and the 3-gene signature as independent prognostic factors for RFS (*p* = 0.017 and *p* = 0.04, respectively) and the 3-gene signature as independent prognostic factor for PFS (*p* = 0.047) (Table 3). Clinical and histological characteristics of the four groups are presented in Supplemental Data 7. The four groups differed in sex, stage, grade and mutational status of *FGFR3.*

**Table 3:** Multivariate analysis of prognostic factors involved in recurrence-free survival and progression-free survival in non-muscle-invasive bladder cancer including the 3-gene signature.

Prognostic factor	Recurrence-free survival		
	Adjusted HR	95% CI	p *
History of NMIBC	2.16	[1.15–4.08]	**0.017**
**3-gene signature (group B vs. AC vs. D)**			**0.04**
Group D	1.00		
Groups A and C	1.70	[1.02–2.84]	
Group B	2.90	[1.05–8.04]	

Prognostic factor	Progression-free survival		
	Adjusted HR	95% CI	p *
T1 stage	2.36	[0.43–12.79]	0.23
High Grade	2.26	[0.20–25.29]	0.51
Cis associated	2.29	[0.41–12.88]	0.35
**3-gene signature (group B vs. AC vs. D)**			**0.047**
Group D	1.00		
Groups A and C	3.50	[1.01–12.09]	
Group B	12.27	[1.03–146.14]	

*HR* hazard ratio,* 95% CI*  95% confidence interval.

*Cox model.

Bold values are statistically significant (*p* < 0.05).

### Response to Bacillus Calmette–Guerin (BCG) therapy

In total, 21/28 (75%) patients with BCG therapy showed recurrent NMIBC or progression to invasive tumor during follow-up, including 15/28 (53.6%) over the first 2 years. Among the 29 investigated genes, only low expression of *FGFR3* was significantly associated with good response to BCG therapy (no or late recurrence) (*p* = 0.037) (Table 4).

**Table 4:** mRNA levels of studied genes in the population who received Bacillus Calmette–Guerin (BCG) therapy and association with response.

GENES	Response^a^ n = 13	No response^b^ n = 15	*p* value**
PVRL4	122.90 [63.41–262.88]*	137.25 [33.37–638.75]*	0.53
MDM4	0.88 [0.50–1.24]	0.98 [0.71–1.63]	0.066
NFE2L2	1.87 [1.06–2.90]	1.59 [0.60–4.13]	0.77
PPARG	14.15 [5.91–18.73]	12.51 [2.26–17.67]	0.17
PIK3CA	1.81 [0.33–9.81]	0.62 [0.37–1.31]	0.39
PRKCI	4.07 [0.00–6.96]	3.71 [0.80–11.16]	0.7
FGFR3	27.94 [9.09–104.57]	73.47 [5.25–143.15]	**0.037**
TACC3	4.98 [1.36–9.32]	3.86 [1.39–12.08]	0.39
FBXW7	0.88 [0.60–1.26]	1.03 [0.64–1.74]	0.44
PAIP1	0.79 [0.63–1.96]	0.85 [0.10–1.09]	0.98
E2F3	2.13 [1.36–2.74]	2.17 [0.81–4.14]	0.73
SOX4	6.39 [3.53–11.76]	6.40 [1.91–12.88]	0.34
EGFR	1.34 [0.45–3.08]	0.92 [0.33–2.67]	0.35
ZNF703	1.09 [0.42–2.18]	1.27 [0.08–3.44]	0.39
PABPC1	2.21 [0.99–4.16]	2.02 [1.38–3.54]	0.27
YWHAZ	1.36 [1.14–3.69]	1.37 [0.68–2.48]	0.61
MYC	0.85 [0.60–1.82]	0.74 [0.30–2.36]	0.56
RXRA	2.04 [1.59–5.79]	1.96 [1.16–4.81]	0.98
GDI2	2.43 [1.50–3.19]	2.12 [0.92–3.10]	0.10
Ki67	8.21 [37.92–72.23]	29.36 [1.80–63.41]	0.27
CCND1	3.40 [0.43–17.88]	2.76 [1.27–10.71]	0.45
HRAS	1.29 [0.77–1.91]	1.30 [0.63–6.00]	0.60
ERBB3	20.49 [11.83–38.13]	19.20 [7.06–43.90]	0.98
MDM2	1.93 [1.17–3.67]	2.06 [0.94–6.08]	0.50
FRS2	0.65 [0.52–0.93]	0.71 [0.48–1.22]	0.24
ERBB2	7.91 [5.15–45.03]	6.43 [2.88–12.50]	0.20
CCNE1	3.78 [2.02–9.72]	3.78 [1.08–27.69]	0.85
ERCC2	1.48 [1.28–2.57]	1.78 [0.80–2.69]	0.45
BCL2L1	1.46 [0.76–1.72]	1.42 [0.75–2.35]	0.98

^a^No recurrence or late recurrence (≥ 24 months).

^b^BCG-refractory or early recurrence (< 24 months).

*Median mRNA value [range].

**Kruskall Wallis H test.

Bold value is statistically significant (*p* < 0.05).

### Association between mRNA expression and survival with MIBC

On univariate analysis, RFS and OS prognosis was poor with high expression of *EGFR* (*p* = 0.045 and *p* = 0.010, respectively) (Supplemental Data 8); RFS prognosis was poor with high expression of *ERCC2* (*p* = 0.025) (Supplemental Data 9); and OS prognosis was poor with low expression of *E2F3* (*p* = 0.030) (Supplemental Data 10).

Multivariate analyses included covariates associated with RFS or OS showing significance at *p* < 0.05 on univariate analysis (i.e. lymph node status and *ERCC2* and *EGFR* status for RFS, and stage, lymph node status and *E2F3* and *EGFR* status for OS). Lymph node status and *ERCC2* status remained associated with RFS (*p* = 0.000014 and *p* = 0.0011) and lymph node status and *E2F3* and *EGFR* level status with OS (*p* = 0.00016, *p* = 0.014 and *p* = 0.035, respectively) (Table 5).

**Table 5:** Multivariate analysis of prognostic factors involved in recurrence-free survival and overall survival in muscle-invasive bladder cancer.

Prognostic factor	Recurrence-free survival		
	HR	95% CI	p *
N + status	4.97	[2.41–10.26]	**0.000014**
ERCC2 overexpression	2.21	[1.20–4.08]	**0.0011**
EGFR overexpression	2.01	[0.97–4.15]	0.061

Prognostic factor	Overall survival		
	HR	95% CI	p *
T3–T4 stage	1.35	[0.61–3.00]	0.46
N + status	4.12	[1.98–8.58]	**0.00016**
E2F3 underexpression	2.98	[1.25–7.09]	**0.014**
EGFR overexpression	2.25	[0.61–3.00]	**0.035**

*HR* hazard ratio,* 95% CI*  95% confidence interval.

*Cox model.

Bold values are statistically significant (*p* < 0.05).

### Protein expression

We chose to assess protein expression of the 2 genes having a significant prognostic impact in NMIBC. The expression of RXRA was nuclear in NMIBC whereas exclusively cytosplasmic in MIBC (Supplemental Data 11). For FGFR3, expression was nuclear in both NMIBC and MIBC. The distribution of the protein expression for NMIBC and MIBC is shown in Supplemental Data 12. There was a statistically significant association between mRNA and protein expression for FGFR3 (*p* < 0.0001) but not for RXRA (Table 6). Protein expression was not associated with prognosis in terms of recurrence or progression of NMIBC (data not shown).

**Table 6:** mRNA level and protein score for RXRA, FGFR3 and CCNE1.

	Protein score				*p* value**
mRNA levels	0 +	1 +	2 +	3 +	
RXRA	1.91 [0.68–2.83]*	1.88 [0.31–5.79]*	1.59 [0.11–9.15]*	3.09 [1.16–5.92]*	0.13
FGFR3	10.40 [0.01–118.70]	61.05 [7.18–143.15]	94.63 [41.84–524.30]	72.13 [58.00–79.54]	** < 0.0001**
CCNE1	9.91 [6.35–13.48]	3.26 [1.64–12.94]	4.85 [0.59–58.55]	4.33 [0.52–17.30]	0.68

*Median mRNA value [range].

**Kruskall-Wallis H-Test.

Bold value is statistically significant (*p* < 0.05).

## Discussion

The analysis of TCGA data for bladder tumors has allowed identifying new oncogenes potentially involved in bladder carcinogenesis because they have activating mutations or focal amplifications. The integrated analysis of the different genetic alterations described in the TCGA has revealed deregulated signaling pathways in bladder tumors and potential preferred therapeutic targets^1,8^. We mainly relied on TCGA data because this study was the gold standard at the time of our study. We aimed to validate some of these TCGA data on a fair-matched cohort of NMIBC and MIBC cases focusing on survival data. Within these 2 populations, the rates of recurrence and/or progression were comparable to those observed typically: half of NMIBC cases show relapse and 35% of pT1 cases progress to muscle-invasive disease. Likewise, more than half of MIBC cases (all stages combined) recur after surgery. In MIBC, we found the same results as in the TCGA data with already known prognostic markers (e.g., *EGFR*). Regarding NMIBC, we found genes of interest such as *FGFR3* but also *RXRA* and *CCNE1*. On multivariate analysis, *RXRA* was associated with RFS but also with PFS. The 3-gene signature highlighted four distinct prognostic groups and was significantly associated with RFS and PFS.

Two studies showed that the transcription factor *RXRA* regulates the expression of *PPARG* and that activating mutation of *RXRA* hyperactivate *PPARG* and could be responsible for nearly 20% of bladder cancer cases^9,10^. These data suggest that *PPARG*, a key gene in bladder cancer development, could be targeted. In this regard, it is noted that association between *PPARG* and *RXRA* mRNA expressions were strong (*p* < 0.0000001 with Spearman’s test). Amplified / overexpressed mutated RXRA-PPARG would modulate the tumor microenvironment and lead to resistance to immunotherapy. Inactivation of PPARG or RXRA would increase sensitivity to immunotherapy^11^. At this time, to our knowledge, there are no clinical trials involving therapies targeting RXRA or PPARG.

Ward et al. studied the mutations of a panel of 23 genes on a cohort of 956 bladder tumors (NMIBC and MIBC) including *TERT* (promoter), *FGFR3*,* PIK3CA*,* ERCC2*,* ERBB2*,* HRAS*,* RXRA*,* KRAS*,* FBXW7*,* ERBB3*,* BRAF* and *NRAS*^12^. *FGFR3* and *HRAS* mutations were of better prognosis (*p* = 0.006 and *p* = 0.04, respectively) and mutations of *RXRA* were significantly associated with a higher risk of recurrence (*p* < 0.05). In our study, we did not find any link between DNA mutations and prognosis. In our cohort, only *RXRA* overexpression was associated with a higher risk of recurrence.

Telomerase Reverse Transcriptase Gene (*TERT*) promoter mutations are recognized as one of the most frequent genetic events across all stages in bladder cancer. Mutations in the core promoter region of TERT cause telomerase reactivation in 60–80% of urothelial bladder tumors^13^. The TERT C228T mutation has been shown to be significantly associated with the prognosis of bladder cancer, particularly with bladder recurrence in NMIBC^14^, whereas TERT C250 mutation appears to be an independent predictive marker of response to BCG treatment^15^. However, in our series, neither C228T nor C250 mutation was associated with prognosis of NMIBC or response to BCG-therapy. Rachakonda et al. showed that the association of mutations with patient survival and disease recurrence may be regulated by a common polymorphism rs2853669 acting as a modifier of the effect of the mutations^16^.

In urothelial carcinomas, the frequency of *FGFR3* mutations varies greatly depending on the type of tumors analyzed^17^. Indeed, the frequency of mutations seems high in pTa and low-grade tumors (about 70%) and much lower in pT1 (30%) or pT2-pT4 and high-grade tumors^1,4^. In our series, the expression of *FGFR3* was stably associated with NMIBC stage, with more frequent overexpression in pTa tumors. mRNA overexpression but not mutations or protein expression were also associated with favorable prognosis. In the literature, immunohistochemical data show protein overexpression also related to good prognosis^18^. In our series, there was a good correlation between mRNA expression and DNA mutation. We have few clinical trial data on targeted anti-FGFR3 therapies in NMIBC. Some MIBC cases also show mutations in *FGFR3* (12%) or *FGFR3/TACC3* translocations (5%)^19^, which suggests a benefit of targeted anti-FGFR3 therapy in these patients. Necchi et al. did not find a prognostic value of *FGFR3* expression in MIBC as compared with NMIBC^20^.

We investigated the response to BCG therapy. Expression of *FGFR3* has been found a BCG response marker, which corroborates the data in mice from Langle et al.^21^. Indeed, in the Langle et al. study, the responder group showed low expression of *FGFR3*, which we also found. Because *FGFR3* is "druggable", anti-*FGFR3* treatments could be combined with BCG therapy to improve the response to treatment. Some studies suggest the addition of such treatments to potentiate the effect of BCG^22,23^, including a phase II trial evaluating dovitinib in BCG-refractory patients with *FGFR3* mutated or not^24^.

*FGFR3* is not the only gene with marked overexpression in NMIBC compared to MIBC. Several genes also appear to be involved in the early stage of bladder carcinogenesis, such as *RXRA*, *PPARG* and *PVRL4*. It is noteworthy that *PVRL4* encodes the nectin-4 adhesion receptor, a new prognostic biomarker and a therapeutic target in various carcinomas. Anti-nectin-4 antibody–drug-conjugate (ADC) seems to have great potential as a therapeutic agent for metastatic urothelial carcinoma^25^. In our series, *PVRL4* was also strongly expressed in NMIBC suggesting that the evaluation of anti-nectin-4 ADC in earlier steps of the disease through clinical trials could be of interest.

*CCNE1* expression is increased in many cancers. As in our study, Rothman et al. showed increased expression of *CCNE1* associated with high grade bladder tumors^26^, which is consistent with its role in cell proliferation. Similarly, in our series, high expression of CCNE1 was associated with poor prognosis in NMIBC.

It is noteworthy that mRNA expression is not always correlated with protein expression, especially for RXRA and CCNE1. Moreover, the protein expression profile appears to be different between NMIBC and MIBC, underlying that NMIBC and MIBC should be considered as two distinct disease entities with different prognostic markers and different putative therapeutic targets.

As regards MIBC, the most attractive gene seems to be *EGFR* because of its prognostic and theragnostic interest. In our study, *EGFR* seemed a prognostic factor for RFS and OS. In the TCGA data, a subgroup of tissue representing poor prognosis, called "basal-like", showed abnormal expression of the *EGFR* pathway^18,26^.The study by Rebouissou et al. suggests that this well-selected group would be a good candidate for anti-EGFR treatment, provided that patients do not have mutations in the RAS pathway that would limit the response to treatment^27^. More recently, anti-EGFR targeted therapies have been evaluated in urothelial carcinomas in a trial demonstrating the benefit of cetuximab combined with paclitaxel as second-line treatment for metastatic bladder cancer^28^.

*ERCC2* is involved in DNA repair. It has recently been studied, in the MIBC as a biomarker of response to neo-adjuvant chemotherapy^29^. Some studies suggest that missenses mutations of *ERCC2* increased cisplatin sensitivity^30–32^. A target population that could benefit the most from this treatment could be identified.

*E2F3*, which was significantly associated with OS in MIBC in our study, belongs to the neuronal subtype^8,33^ that is characterized by the expression of both neuroendocrine and neuronal genes reflecting a proliferative state.

Taking into account the prognostic impact and the predictive value of these two genes, ERCC2 and E2F3 expression levels may probably allow to guide the indication of chemotherapy in neoadjuvant or metastatic setting.

The strengths of our study lie in the robustness of the material, which is a well-defined cohort of both NMIBC and MIBC with strong clinical follow-up. However, there are some weaknesses notably its retrospective design and incomplete analysis of DNA mutations and protein expression levels since only focusing on target genes.

In conclusion, regarding the prognosis of NMIBC, predicting the risk of progression is difficult and the identification of markers is particularly interesting. Our results on *RXRA* and *FGFR3* and the 3-gene signature, if they are confirmed, could allow for adapting the management by intensifying the surveillance and/or proposing early cystectomy in patients with abnormal expression associated with poor prognosis. It may be an interesting approach to include other omics platforms like metabolomics, proteomics, lipidomics to validate the three genes for prognosis.

## Methods

### Patients and samples

We analyzed 128 urothelial carcinoma samples from patients who had undergone transurethral bladder resection or radical cystectomy in our hospital between 2002 and 2007. Specimens of normal bladder tissue from 21 patients undergoing surgery unrelated to bladder tumors (transurethral resection of the prostate or prostatic adenomectomy) were used as normal bladder tissue. All patients gave their signed informed consent. This study received approval from an institutional review board (the Research Ethics Committee of Paris Descartes University) and was conducted according to the principles outlined in the Declaration of Helsinki.

Immediately after surgery, tumor samples were frozen in liquid nitrogen and stored at − 80 °C (for RNA extraction) and fixed in formaldehyde. Each tumor was reviewed by 2 pathologists (DD and MS) who were blinded to clinical outcomes. Tumors were re-staged according to the 2009 American Joint Committee on TNM classification of bladder tumors and graded according to the 2004 World Health Organization grading scheme^34,35^.

Complete clinical, histological and survival data were available for these 128 patients (107 men and 21 women; median age 70 years [range: 31–91]). Pathological staging revealed NMIBC in 61 (25 low-grade pTa, 14 high-grade pTa, 22 high-grade pT1) and high-grade MIBC in 67. For NMIBC patients, the median follow-up was 58 months (range: 3–158 months; mean follow-up, 64 months). For MIBC patients, the median follow-up was 12 months (range: 1–152 months; mean follow-up: 31 months). Clinical, histological and survival characteristics are presented in Table 1.

### Gene selection

Recent findings from the genome-wide analysis of TCGA data highlighted new oncogenes potentially involved in bladder carcinogenesis because of activating mutations [*PIK3CA* (20%), *ERCC2* (12%), *FGFR3* (12%), *ERBB3* (11%), *FBXW7* (10%), *RXRA* (9%), *NFE2L2* (8%), *FRS2* (8%)] or recurrent amplifications [*YWHAZ* (22%), *E2F3* (20%), *SOX4* (20%), *PVRL4* (19%), *PPARG* (17%), *PABPC1* (17%), *MYC* (13%), *CCNE1* (12%), *BCL2L1* (11%), *EGFR* (11%), *CCND1* (10%), *ZNF703* (10%), *GDI2* (10%), *MDM2* (9%), PIK3CA (8%)] or overexpression [*PVRLA4* (26%), *PABPC1* (26%), *YWHAZ* (26%), *SOX4* (25%), *E2F3* (24%), *GDI2* (21%), *BCL2L1* (21%), *PAIP1* (19%), *EGFR* (18%), *FRS2* (18%), *ZNF703* (18%), *CCNE1* (17%), *PRKCI* (14%), *PIK3CA* (13,5%), *ERBB3* (12%), *PPARG* (12%), *CCND1* (11%), *ERBB2* (10%), *MDM2* (9%), *ERCC2* (8,7%)]^1^. In addition to TCGA data, recent articles have highlighted previously unknown genetic alterations in bladder cancer. *MDM4* was described as a prognostic marker of recurrence in NMIBC^5^. *TACC3* and *FGFR3* can form a fusion gene, described in some cancers^1,4,6^. In addition, the role of the RAS family has been extensively studied in oncology. *HRAS* is mutated in 5% of bladder cancers. Analysis of cell lines of bladder cancer cases revealed that this population could be treated with anti-MEK and -mTOR therapy^7^. In the same way, the MYC proto-oncogene is potentially "drugable"; it has been found a prognostic factor of NMIBC recurrence^2^.

Finally, we selected 29 genes (Supplemental Data 13) for analysis. We chose one endogenous RNA control gene to RT-PCR analysis, namely *TBP* (GenBank accession No. NM_003194), which encodes the TATA box-binding protein.

### DNA mutation analysis

We analyzed *HRAS*, *FGFR3*,* PIK3CA* and *TERT* which have well-described mutational spots (codons 12–13 and 61 for *HRAS*; exon 7 (codons 248–249) and exon 10 (codons 372–373-375–382) for *FGFR3*; exons 9 and 20 for *PIK3CA*; C228T and C250T mutations for *TERT*). The assessment involved a screening with high-resolution melt (HRM) analysis followed by Sanger sequencing of samples with a mutated profile on HRM to validate the HRM data and to determine the nomenclature of mutations found.

### Real-time quantitative RT-PCR

The theoretical basis, primers and PCR consumables, RNA extraction, cDNA synthesis, and PCR reaction conditions have been previously described in detail^36^. Each sample was normalized to *TBP* level. N*target* values for samples were normalized such that the median of the 21 normal-bladder Ntarget values was 1. For each investigated gene, mRNA values ≥ 3 were considered to represent overexpression and ≤ 0.33 under-expression. We previously used the same cut-off value for altered tumor gene expression^36^. We also used the optimal cut-off calculated by the area under the receiver operating characteristic curve for prognostic studies.

### Analysis of protein expression

Representative blocks of paraffin-embedded tumor samples were available. For each tumor, 2 observers, including at least 1 expert pathologist, selected the tumor block. The area of interest was defined as the one with the highest stage and grade tumor area. A table is then arranged to guide the reading of the Tissu-Micro-Array (TMA). The removal of the tissue in the area of interest is done using a trocar of the Tissu-Micro-Arrayer machine (Alphelys) and is placed on a blank paraffin block according to the plane defined beforehand on paper. Positive control tissues are also deposited and will be used when reading the TMA. Finally, the spots are fixed by depositing the block thus made on a hot plate (37–40° C). Briefly, serial 3-µm tissue sections were made using a paraffin microtome (Microtome Micron AM 360) and then were deparaffinized, rehydrated and pretreated in appropriate buffer for antigen retrieval by using a Leica automat. Tissue slides were then incubated at 48 °C with a primary antibody, anti-RXRA (ab191176, Abcam) or anti-FGFR3 (sc-13121, Santa Cruz); (both 1:100), then appropriate secondary antibodies.

We used a semi-quantitative analysis of protein expression with the following scores: 0 (no positive cells), 1 + (few positive cells), 2 + (numerous positive cells) and 3 + (a lot of positive cells). All quantification was performed with blinding to patient status by an expert pathologist (DD).

### Statistical analysis

The clinicopathological features of NMIBC and MIBC cases were tested for their association with tumor recurrence and survival by using chi-square test for categorical variables. The associations between clinical and histological variables and mRNA levels were tested by the non-parametric Mann–Whitney U and Kruskal–Wallis H tests. Unsupervised hierarchical cluster analyses involved use of the WARD algorithm to identify homogenous tumor groups in terms of molecular data. Survival curves were derived by the Kaplan–Meier method, with the log-rank test used to compare survival between groups. Cox proportional-hazards regression was used to estimate hazard ratios (HRs) and their 95% confidence intervals (95% CIs) for covariates associated with RFS, PFS or OS showing significance at *p* < 0.05 on univariate analysis. Differences between two populations were considered statistically significant at confidence levels > 95% (*p* < 0.05).

## Supplementary information

Supplementary Information 1.

Supplementary Information 2.

Supplementary Information 3.

Supplementary Information 4.

Supplementary Information 5.

Supplementary Information 6.

Supplementary Information 7.

Supplementary Information 8.

Supplementary Information 9.

Supplementary Information 10.

Supplementary Information 11.

Supplementary Information 12.

Supplementary Information 13.

## Data Availability

Materials and data are available.

## Abbreviations

BCG: Bacillus Calmette–Guerin
cDNA: Complementary deoxyribonucleic acid
IHC: Immunohistochemistry
NMIBC: Non-muscle-invasive bladder cancer
MIBC: Muscle-invasive bladder cancer
mRNA: Messenger ribonucleic acid
OS: Overall survival
PFS: Progression-free survival
RFS: Recurrence-free survival
RT-PCR: Reverse-transcriptase polymerase chain reaction
TCGA: The Cancer Genome Atlas
TNM: Tumor-node metastasis
