# Supplementary material for: Assessment of prognostic implication of a panel of oncogenes in bladder cancer and identification of a 3-gene signature associated with recurrence and progression risk in non-muscle-invasive bladder cancer
Source: Sci Rep. 2020 Oct 6;10:16641. doi: 10.1038/s41598-020-73642-8 (PMC7538919; doi:10.1038/s41598-020-73642-8)
Supplement: Supplementary file 1 — Supplementary Information 1. [file 41598_2020_73642_MOESM1_ESM.docx]

**ASSESSMENT OF PROGNOSTIC IMPLICATION OF A PANEL OF ONCOGENES IN BLADDER CANCER AND IDENTIFICATION OF A 3-GENE SIGNATURE ASSOCIATED WITH RECURRENCE AND PROGRESSION RISK IN NON-MUSCLE-INVASIVE BLADDER CANCER_**Le Goux Constance, Vacher Sophie, Schnitzler Anne, Barry Delongchamps Nicolas, Zerbib Marc, Peyromaure Michaël, Mathilde Sibony, Yves Allory, Bieche Ivan**,** Damotte Diane, Pignot Géraldine

**Suppl. data 1: Frequency of mutations for *HRAS*, *FGFR3*, *PIK3CA* and *TERT***

**a) Frequency of *HRAS* mutations**

| **HRAS** | **Codon 12-13**  n (%) | **Codon 61**  n (%) | **Total**  n (%) |
| --- | --- | --- | --- |
| **All tumors (n=103)** | 3 (2.9) | 0 (0.0) | 3 (2.9) |
| **NMIBC (n=44)** | 2 (4.5) | 0 (0.0) | 2 (4.5) |
| **MIBC (n=59)** | 1 (1.7) | 0 (0.0) | 1 (1.7) |

**b) Frequency of *FGFR3* mutations**

| **FGFR3** | **Total**  n (%) |
| --- | --- |
| **All tumors (n=103)** | 28 (27.2) |
| **NMIBC (n=44)** | 22 (50.0) |
| **MIBC (n=59)** | 6 (10.2) |

**c) Frequency of *PIK3CA* mutations**

| **PIK3CA** | **Exon 9**  n (%) | **Exon 20**  n (%) | **Total**  n (%) |
| --- | --- | --- | --- |
| **All tumors (n=127)** | 13 (10.2) | 6 (4.7) | 19 (14.9) |
| **NMIBC (n=60)** | 10 (16.7) | 0 (0.0) | 10 (16.7) |
| **MIBC (n=67)** | 3 (4.5) | 6 (9.0) | 9 (13.5) |

**c) Frequency of *TERT* mutations**

| **TERT** | **C228 mutation**  n (%) | **C250 mutation**  n (%) | **Total**  n (%) |
| --- | --- | --- | --- |
| **All tumors (n=103)** | 65 (63.1) | 14 (13.6) | 79 (76.7) |
| **NMIBC (n=44)** | 28 (63.6) | 7 (15.9) | 35 (79.5) |
| **MIBC (n=59)** | 37 (62.7) | 7 (11.9) | 44 (74.6) |
