# Supplementary material for: Assessment of prognostic implication of a panel of oncogenes in bladder cancer and identification of a 3-gene signature associated with recurrence and progression risk in non-muscle-invasive bladder cancer
Source: Sci Rep. 2020 Oct 6;10:16641. doi: 10.1038/s41598-020-73642-8 (PMC7538919; doi:10.1038/s41598-020-73642-8)
Supplement: Supplementary file 2 — Supplementary Information 2. [file 41598_2020_73642_MOESM2_ESM.doc]

**ASSESSMENT OF PROGNOSTIC IMPLICATION OF A PANEL OF ONCOGENES IN BLADDER CANCER AND IDENTIFICATION OF A 3-GENE SIGNATURE ASSOCIATED WITH RECURRENCE AND PROGRESSION RISK IN NON-MUSCLE-INVASIVE BLADDER CANCER_**Le Goux Constance, Vacher Sophie, Schnitzler Anne, Barry Delongchamps Nicolas, Zerbib Marc, Peyromaure Michaël, Mathilde Sibony, Yves Allory, Bieche Ivan**,** Damotte Diane, Pignot Géraldine

**Suppl. data 2: mRNA expression of the 29 studied genes in normal and tumor bladder samples**

| **GENES** | **Normal (n=21)** | **NMIBC (n=61)** | **Underexpression^a^  n (%)** | **Overexpression^b^  n (%)** | **MIBC (n=67)** | **Underexpression^a^  n (%)** | **Overexpression^b^  n (%)** | ***MIBC/ NMIBC  p*-value**** | **AUC ^£^** | ***NMIBC/N  p*-value**** | **AUC ^£^** | ***MIBC/N  p*-value**** | **AUC ^£^** |
| --- | --- | --- | --- | --- | --- | --- | --- | --- | --- | --- | --- | --- | --- |
| **PVRL4** | 1.00 [0.00-34.48]* | 144.81 [33.37-751.90]* | 0 (0.0) | 61 (100.0) | 70.26 [0.00-820.53]* | 2 (3.0) | 61 (91.0) | **0.00000022** | 0.234 | **<10^-7^** | 0.998 | **0.00000015** | 0.882 |
| **MDM4** | 1.00 [0.59-2.32] | 0.98 [0.50-2.21] | 0 (0.0) | 0 (0.0) | 0.80 [0.26-1.72] | 1 (1.5) | 0 (0.0) | **0.00049** | 0.327 | 0.86 | 0.487 | **0.0079** | 0.307 |
| **NFE2L2** | 1.00 [0.32-2.35] | 2.00 [0.60-6.58] | 0 (0.0) | 11 (18.0) | 1.58 [0.47-6.17] | 0 (0.0) | 5 (7.5) | **0.0026** | 0.345 | **0.00018** | 0.851 | **0.0035** | 0.712 |
| **PPARG** | 1.00 [0.91-1.86] | 25.02 [4.51-55.73] | 0 (0.0) | 61 (100.0) | 8.94 [0.13-174.90] | 3 (4.5) | 41 (61.2) | **<10^-7^** | 0.169 | **<10^-7^** | 0.993 | **0.012** | 0.682 |
| **PIK3CA** | 1.00 [0.37-2.29] | 0.54 [0.21-2.01] | 1 (1.6) | 0 (0.0) | 0.67 [0.32-2.90] | 1 (1.5) | 0 (0.0) | **0.00052** | 0.678 | **0.0000014** | 0.146 | **0.000037** | 0.200 |
| **PRKCI** | 1.00 [0.02-4.21] | 4.01 [0.00-12.39] | 1 (1.6) | 48 (78.7) | 4.16 [1.76-20.08] | 0 (0.0) | 53 (79.1) | 0.29 | 0.555 | **<10^-7^** | 0.941 | **<10^-7^** | 0.967 |
| **FGFR3** | 1.00 [0.65-2.41] | 25.76 [0.89-187.25] | 0 (0.0) | 57 (93.4) | 2.56 [0.00-47.52] | 14 (20.9) | 32 (47.8) | **<10^-7^** | 0.148 | **<10^-7^** | 0.987 | **0.00044** | 0.755 |
| **TACC3** | 1.00 [0.23-18.12] | 3.97 [0.69-15.59] | 0 (0.0) | 40 (65.6) | 7.80 [0.90-22.20] | 0 (0.0) | 58 (86.6) | **0.0000049** | 0.734 | **0.0000018** | 0.851 | **<10^-7^** | 0.914 |
| **FBXW7** | 1.00 [0.61-2.05] | 1.06 [0.57-2.01] | 0 (0.0) | 0 (0.0) | 1.07 [0.42-3.16] | 0 (0.0) | 1 (1.5) | 0.23 | 0.562 | 0.88 | 0.489 | 0.65 | 0.533 |
| **PAIP1** | 1.00 [0.80-2.10] | 0.85 [0.10-1.96] | 1 (1.6) | 0 (0.0) | 1.02 [0.46-3.43] | 0 (0.0) | 2 (3.0) | **0.00057** | 0.677 | **0.000012** | 0.179 | **<10^-7^** | 0.453 |
| **E2F3** | 1.00 [0.18-2.09] | 2.22 [0.81-4.14] | 0 (0.0) | 10 (16.4) | 2.41 [0.75-13.46] | 0 (0.0) | 24 (35.8) | 0.23 | 0.561 | **<10^-7^** | 0.953 | **<10^-7^** | 0.934 |
| **SOX4** | 1.00 [0.61-1.07] | 2.55 [0.76-5.15] | 0 (0.0) | 19 (31.1) | 2.10 [0.31-14.79] | 1 (1.5) | 20 (29.9) | 0.44 | 0.461 | **<10^-7^** | 0.981 | **<10^-7^** | 0.958 |
| **EGFR** | 1.00 [0.28-1.89] | 0.99 [0.20-3.08] | 2 (3.3) | 1 (1.6) | 1.29 [0.01-8.64] | 5 (7.5) | 11 (16.4) | **0.025** | 0.615 | 0.88 | 0.511 | **0.049** | 0.643 |
| **ZNF703** | 1.00 [0.34-4.48] | 1.07 [0.08-10.23] | 5 (8.2) | 7 (11.5) | 0.60 [0.10-13.45] | 12 (17.9) | 4 (6.0) | **0.0024** | 0.344 | 0.63 | 0.535 | **0.035** | 0.347 |
| **PABPC1** | 1.00 [0.40-3.91] | 2.18 [0.99-4.51] | 0 (0.0) | 9 (14.8) | 1.21 [0.26-11.13] | 1 (1.5) | 9 (13.4) | **0.0000085** | 0.272 | **<10^-7^** | 0.898 | 0.13 | 0.609 |
| **YWHAZ** | 1.00 [0.76-3.44] | 1.38 [0.68-3.69] | 0 (0.0) | 5 (8.2) | 1.45 [0.46-7.84] | 0 (0.0) | 8 (11.9) | 0.74 | 0.517 | **0.0013** | 0.736 | **0.0021** | 0.723 |
| **MYC** | 1.00 [0.35-8.88] | 1.01 [0.08-3.68] | 3 (4.9) | 3 (4.9) | 1.58 [0.10-8.05] | 3 (4.5) | 10 (14.9) | **0.0015** | 0.663 | 0.56 | 0.543 | **0.016** | 0.675 |
| **RXRA** | 1.00 [0.49-2.29] | 2.42 [0.41-9.23] | 0 (0.0) | 20 (32.8) | 1.41 [0.11-9.15] | 2 (3.0) | 10 (14.9) | **<10^-7^** | 0.227 | **<10^-7^** | 0.930 | **0.044** | 0.646 |
| **GDI2** | 1.00 [0.16-2.48] | 2.26 [0.92-3.44] | 0 (0.0) | 9 (14.8) | 2.42 [1.10-5.59] | 0 (0.0) | 19 (28.4) | 0.29 | 0.554 | **<10^-7^** | 0.948 | **<10^-7^** | 0.949 |
| **Ki67** | 1.00 [0.00-93.03] | 33.85 [1.45-84.55] | 0 (0.0) | 57 (93.4) | 53.80 [2.39-197.75] | 0 (0.0) | 66 (98.5) | **0.000019** | 0.719 | **<10^-7^** | 0.923 | **<10^-7^** | 0.947 |
| **CCND1** | 1.00 [0.48-2.16] | 3.34 [0.18-19.98] | 2 (3.3) | 35 (67.4) | 1.37 [0.10-31.80] | 11 (16.4) | 11 (16.4) | **0.00000011** | 0.228 | **<10^-7^** | 0.901 | 0.25 | 0.638 |
| **HRAS** | 1.00 [0.48-2.10] | 1.44 [0.63-6.00] | 0 (0.0) | 3 (4.9) | 1.27 [0.35-7.47] | 0 (0.0) | 5 (7.5) | **0.037** | 0.393 | **0.00018** | 0.775 | 0.057 | 0.638 |
| **ERBB3** | 1.00 [0.38-1.28] | 2.36 [0.54-8.77] | 0 (0.0) | 11 (18.0) | 1.07 [0.00-10.89] | 13 (19.4) | 5 (7.5) | **<10^-7^** | 0.19 | **<10^-7^** | 0.993 | **0.00000094** | 0.856 |
| **MDM2** | 1.00 [0.59-1.59] | 2.01 [0.94-11.42] | 0 (0.0) | 12 (19.7) | 1.39 [0.38-13.63] | 0 (0.0) | 9 (13.4) | **0.00019** | 0.309 | **<10^-7^** | 0.936 | **0.0044** | 0.707 |
| **FRS2** | 1.00 [0.60-1.91] | 0.71 [0.40-27.38] | 0 (0.0) | 1 (1.6) | 0.78 [0.34-6.53] | 0 (0.0) | 1 (1.5) | 0.74 | 0.517 | **0.0000056** | 0.166 | **0.0016** | 0.271 |
| **ERBB2** | 1.00 [0.95-1.37] | 2.73 [0.81-17.32] | 0 (0.0) | 24 (39.3) | 1.35 [0.01-110.73] | 9 (1.4) | 18 (26.9) | **0.00023** | 0.311 | **<10^-7^** | 0.979 | **0.000027** | 0.805 |
| **CCNE1** | 1.00 [0.70-1.21] | 7.55 [1.98-55.39] | 0 (0.0) | 58 (95.1) | 11.30 [1.04-117.10] | 0 (0.0) | 60 (89.6) | **0.04** | 0.605 | **<10^-7^** | 0.970 | **<10^-7^** | 0.938 |
| **ERCC2** | 1.00 [0.31-2.47] | 1.78 [0.52-6.10] | 0 (0.0) | 8 (13.1) | 1.71 [0.71-6.03] | 0 (0.0) | 8 (11.9) | 0.61 | 0.474 | **0.000006** | 0.833 | **0.000035** | 0.801 |
| **BCL2L1** | 1.00 [0.48-1.93] | 1.42 [0.68-4.45] | 0 (0.0) | 2 (3.3) | 1.55 [0.39-6.23] | 0 (0.0) | 5 (7.5) | 0.26 | 0.558 | **0.00069** | 0.749 | **0.00054** | 0.751 |

N=normal

* Median value [range]

** Kruskal Wallis H test

£ Area under the receiver operating characteristic curve (AUC)
