# Supplementary material for: Assessment of prognostic implication of a panel of oncogenes in bladder cancer and identification of a 3-gene signature associated with recurrence and progression risk in non-muscle-invasive bladder cancer
Source: Sci Rep. 2020 Oct 6;10:16641. doi: 10.1038/s41598-020-73642-8 (PMC7538919; doi:10.1038/s41598-020-73642-8)
Supplement: Supplementary file 3 — Supplementary Information 3. [file 41598_2020_73642_MOESM3_ESM.docx]

**ASSESSMENT OF PROGNOSTIC IMPLICATION OF A PANEL OF ONCOGENES IN BLADDER CANCER AND IDENTIFICATION OF A 3-GENE SIGNATURE ASSOCIATED WITH RECURRENCE AND PROGRESSION RISK IN NON-MUSCLE-INVASIVE BLADDER CANCER_**Le Goux Constance, Vacher Sophie, Schnitzler Anne, Barry Delongchamps Nicolas, Zerbib Marc, Peyromaure Michaël, Mathilde Sibony, Yves Allory, Bieche Ivan**,** Damotte Diane, Pignot Géraldine

**Suppl. data 3: mRNA expression of the 29 studied genes in the NMIBC subgroups**

|  | **All NMIBC tumors**  **(n=61)** | **pTa low grade**  **(n=25)** | **pTa high grade**  **(n=14)** | **pT1 high grade**  **(n=22)** | **p****  **pTa versus pT1** | **p****  **low versus high grade** |
| --- | --- | --- | --- | --- | --- | --- |
| PVRL4 | 144.81 [33.37-751.90]* | 126.08 [54.94-440.42]* | 226.62 [104.72-638.75]* | 141.03 [33.37-751.90]* | 0.60 | 0.05 |
| MDM4 | 0.98 [0.50-2.21] | 1.04 [0.71-2.00] | 1.08 [0.57-1.77] | 0.88 [0.50-2.21] | 0.20 | 0.88 |
| NFE2L2 | 2.00 [0.60-6.58] | 1.97 [0.99-3.90] | 2.23 [1.06-3.74] | 2.09 [0.60-6.58] | 0.98 | 0.58 |
| PPARG | 25.02 [4.51-55.73] | 25.44 [11.82-37.46] | 25.73 [12.89-55.73] | 24.38 [4.51-35.34] | 0.28 | 0.55 |
| PIK3CA | 0.54 [0.21-2.01] | 0.54 [0.21-1.11] | 0.51 [0.35-1.51] | 0.62 [0.38-2.01] | **0.03** | 0.17 |
| PRKCI | 4.01 [0.00-12.39] | 3.68 [0.00-7.57] | 4.03 [1.54-11.16] | 4.51 [0.80-12.39] | 0.14 | 0.15 |
| FGFR3 | 25.76 [0.89-187.25] | 27.91 [3.25-79.68] | 30.55 [0.89-68.89] | 17.98 [1.40-187.25] | **0.03** | 0.17 |
| TACC3 | 3.97 [0.69-15.59] | 2.39 [0.69-12.08] | 4.76 [1.36-13.43] | 6.63 [1.39-15.59] | **0.008** | **0.001** |
| FBXW7 | 1.06 [0.57-2.01] | 0.94 [0.57-1.46] | 1.08 [0.60-1.40] | 0.19 [0.64-2.01] | **0.04** | **0.03** |
| PAIP1 | 0.85 [0.10-1.96] | 0.80 [0.58-1.48] | 0.81 [0.10-1.09] | 0.84 [0.22-1.80] | 0.09 | 0.28 |
| E2F3 | 2.22 [0.81-4.14] | 2.12 [0.81-3.25] | 2.23 [0.16-6.65] | 0.90 [0.43-1.96] | 0.27 | **0.004** |
| SOX4 | 2.55 [0.76-5.15] | 1.73 [0.88-4.20] | 2.67 [1.24-5.15] | 2.84 [0.76-4.71] | **0.04** | **0.007** |
| EGFR | 0.99 [0.20-3.08] | 0.84 [0.39-2.07] | 0.80 [0.33-1.74] | 1.46 [0.20-3.08] | 0.06 | 0.35 |
| ZNF703 | 1.07 [0.08-10.23] | 0.86 [0.21-3.08] | 1.52 [0.57-3.79] | 0.94 [0.08-10.23] | 0.96 | **0.03** |
| PABPC1 | 2.18 [0.99-4.51] | 2.16 [0.99-3.54] | 2.31 [1.43-3.68] | 2.12 [1.26-4.51] | 0.55 | 0.24 |
| YWHAZ | 1.38 [0.68-3.69] | 1.29 [0.83-2.48] | 1.51 [0.68-1.93] | 1.69 [0.75-3.69] | **0.01** | **0.002** |
| MYC | 1.01 [0.68-3.08] | 0.94 [0.29-1.82] | 1.17 [0.36-3.40] | 0.92 [0.08-3.68] | 0.78 | 0.13 |
| RXRA | 2.42 [0.41-9.23] | 2.42 [0.41-5.82] | 2.45 [1.16-9.23] | 2.31 [0.79-5.79] | 0.57 | 0.72 |
| GDI2 | 2.26 [0.92-3.44] | 2.25 [1.28-3.44] | 2.41 [1.13-3.07] | 2.22 [0.92-3.23] | **0.01** | 0.59 |
| KI67 | 33.85 [1.45-84.55] | 17.64 [1.45-75.16] | 38.23 [6.96-50.00] | 35.67 [1.80-84.55] | **0.01** | **0.002** |
| CCND1 | 3.34 [0.18-19.98] | 3.32 [0.43-19.98] | 3.13 [1.27-10.71] | 3.43 [0.18-17.88] | 0.72 | 0.71 |
| HRAS | 1.44 [0.63-6.00] | 1.63 [0.97-1.87] | 1.45 [0.63-2.81] | 1.37 [0.67-6.00] | 0.70 | 0.58 |
| ERBB3 | 2.36 [0.54-8.77] | 2.08 [0.54-3.90] | 2.45 [1.07-5.49] | 2.45 [0.88-8.77] | 0.92 | 0.38 |
| MDM2 | 2.01 [0.94-11.42] | 1.70 [1.21-3.67] | 1.97 [1.09-11.42] | 2.31 [0.94-7.50] | 0.16 | 0.18 |
| FRS2 | 0.71 [0.40-27.38] | 0.71 [0.40-0.96] | 0.70 [0.59-1.22] | 0.73 [0.52-27.38] | 0.76 | 0.59 |
| ERBB2 | 2.73 [0.81-17.32] | 2.55 [1.06-4.97] | 3.16 [1.01-6.85] | 2.70 [0.81-17.32] | 0.9 | 0.17 |
| CCNE1 | 7.55 [1.98-55.39] | 5.06 [1.98-28.20] | 8.84 [4.02-23.54] | 9.38 [2.15-55.39] | 0.08 | **0.01** |
| ERCC2 | 1.78 [0.52-6.10] | 1.57 [0.52-3.24] | 2.11 [1.09-4.17] | 1.81 [1.27-6.10] | 0.42 | **0.02** |
| BCL2L1 | 1.42 [0.68-4.45] | 1.30 [0.70-2.02] | 1.44 [0.84-2.41] | 1.69 [0.68-4.45] | **0.04** | 0.07 |

*Median mRNA value [range]

** Kruskal Wallis H Test
