# Supplementary material for: Assessment of prognostic implication of a panel of oncogenes in bladder cancer and identification of a 3-gene signature associated with recurrence and progression risk in non-muscle-invasive bladder cancer
Source: Sci Rep. 2020 Oct 6;10:16641. doi: 10.1038/s41598-020-73642-8 (PMC7538919; doi:10.1038/s41598-020-73642-8)
Supplement: Supplementary file 4 — Supplementary Information 4. [file 41598_2020_73642_MOESM4_ESM.docx]

**ASSESSMENT OF PROGNOSTIC IMPLICATION OF A PANEL OF ONCOGENES IN BLADDER CANCER AND IDENTIFICATION OF A 3-GENE SIGNATURE ASSOCIATED WITH RECURRENCE AND PROGRESSION RISK IN NON-MUSCLE-INVASIVE BLADDER CANCER_**Le Goux Constance, Vacher Sophie, Schnitzler Anne, Barry Delongchamps Nicolas, Zerbib Marc, Peyromaure Michaël, Mathilde Sibony, Yves Allory, Bieche Ivan**,** Damotte Diane, Pignot Géraldine

**Suppl. data 4: Recurrence-free and progression-free survival according to mRNA level of *RXRA* in non-muscle-invasive bladder cancer (NMIBC)**

**a) Recurrence-free survival by mRNA level of *RXRA* in NMIBC**


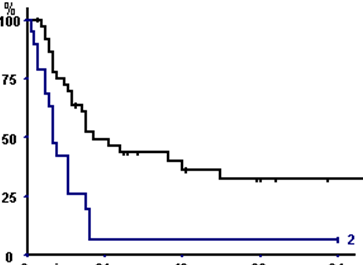


**0 24 48 72 96 months**

***RXRA* low expression**

**p = 0.0017**

***RXRA* high expression**

**b) Progression-free survival by mRNA expression of *RXRA* in NMIBC**


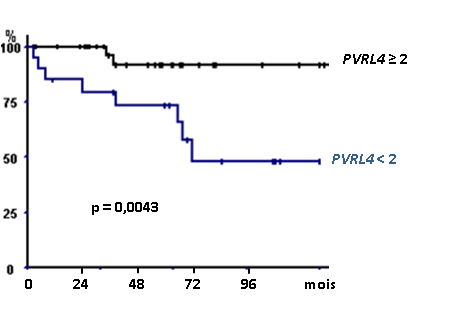


**0 24 48 72 96 months**

***RXRA* low expression**

***RXRA* high expression**

**p = 0.0043**
