# Supplementary material for: Assessment of prognostic implication of a panel of oncogenes in bladder cancer and identification of a 3-gene signature associated with recurrence and progression risk in non-muscle-invasive bladder cancer
Source: Sci Rep. 2020 Oct 6;10:16641. doi: 10.1038/s41598-020-73642-8 (PMC7538919; doi:10.1038/s41598-020-73642-8)
Supplement: Supplementary file 7 — Supplementary Information 7. [file 41598_2020_73642_MOESM7_ESM.doc]

**ASSESSMENT OF PROGNOSTIC IMPLICATION OF A PANEL OF ONCOGENES IN BLADDER CANCER AND IDENTIFICATION OF A 3-GENE SIGNATURE ASSOCIATED WITH RECURRENCE AND PROGRESSION RISK IN NON-MUSCLE-INVASIVE BLADDER CANCER_**Le Goux Constance, Vacher Sophie, Schnitzler Anne, Barry Delongchamps Nicolas, Zerbib Marc, Peyromaure Michaël, Mathilde Sibony, Yves Allory, Bieche Ivan**,** Damotte Diane, Pignot Géraldine

Suppl. data 7: Clinical and pathological characteristics of the four NMIBC tumor groups generated after classification with the 3-gene signature (*RXRA*, *FGFR3* and *CCNE1*).

|  |  | | **Group A** | | **Group B** | | **Group C** | | **Group D** | |  | |
| --- | --- | --- | --- | --- | --- | --- | --- | --- | --- | --- | --- | --- |
|  |  | | **n (%)** | | **n (%)** | | **n (%)** | | **n (%)** | | **p** * | |
| **Total population** | | 15 (100) | | 15 (100) | | 16 (100) | | 15 (100) | |  | |  |
| **Age (years)** | ≥60  <60 | 12 (80.0)  3 (20.0) | | 14 (93.3)  1 (6.7) | | 10 (62.5)  6 (37.5) | | 9 (60.0)  6 (40.0) | | 0.12 | |  |
| **Sex** | Male | 14 (93.3) | | 15 (100.0) | | 11 (68.8) | | 14 (93.3) | | **0.032** | |  |
|  | Female | 1 (6.7) | | 0 (0.0) | | 5 (31.2) | | 1 (6.7) | |  | |  |
| **Smoking status** | Non-smoker | 9 (60.0) | | 5 (33.3) | | 5 (31.2) | | 8 (53.3) | | 0.28 | |  |
|  | Smoker | 6 (40.0) | | 10 (66.7) | | 11 (68.8) | | 7 (46.7) | |  | |  |
| **History of NMIBC** | No | 7 (46.7) | | 7 (46.7) | | 8 (50.0) | | 3 (20.0) | | 0.30 | |  |
|  | Yes | 8 (53.3) | | 8 (53.3) | | 8 (50.0) | | 12 (80.0) | |  | |  |
| **Cis associated** | No | 14 (93.3) | | 13 (86.7) | | 16 (100.0) | | 15 (100.0) | | 0.26 | |  |
|  | Yes | 1 (6.7) | | 2 (13.3) | | 0 (0.0) | | 0 (0.0) | |  | |  |
| **Grade** | Low | 8 (53.3) | | 1 (6.7) | | 6 (37.5) | | 10 (66.7) | | **0.006** | |  |
|  | High | 7 (46.7) | | 14 (93.3) | | 10 (62.5) | | 5 (33.3) | |  | |  |
| **Tumor stage** | Ta | 4 (26.7) | | 5 (33.3) | | 12 (75.0) | | 4 (26.7) | | **0.014** | |  |
|  | T1 | 11 (73.3) | | 10 (66.7) | | 4 (25.0) | | 11 (73.3) | |  | |  |
| ***HRAS* mutation^£^** | No | 13 (100.0) | | 11 (91.7) | | 8 (100.0) | | 10 (90.9) | | 0.39 | |  |
|  | Yes | 0 (0.0) | | 1 (8.3) | | 0 (0.0) | | 1 (9.1) | |  | |  |
| ***FGFR3* mutation^£^** | No | 6 (46.2) | | 10 (83.3) | | 3 (37.5) | | 3 (27.2) | | **0.042** | |  |
|  | Yes | 7 (53.8) | | 2 (16.7) | | 5 (62.5) | | 8 (72.3) | |  | |  |
| ***PIK3CA* mutation^££^** | No | 13 (86.7) | | 12 (80.0) | | 13 (81.3) | | 12 (85.7) | | 0.95 | |  |
|  | Yes | 2 (13.3) | | 3 (20.0) | | 3 (18.7) | | 2 (14.3) | |  | |  |
| ***TERT* mutation^£^** | No | 2 (15.4) | | 1 (8.3) | | 2 (25.0) | | 4 (36.4) | | 0.38 | |  |
|  | Yes | 11 (84.6) | | 11 (91.7) | | 6 (75.0) | | 7 (63.6) | |  | |  |

*chi-square test

**^£^** Information available for 44 patients; **^££^** Information available for 60 patients
