# Supplementary material for: Assessment of prognostic implication of a panel of oncogenes in bladder cancer and identification of a 3-gene signature associated with recurrence and progression risk in non-muscle-invasive bladder cancer
Source: Sci Rep. 2020 Oct 6;10:16641. doi: 10.1038/s41598-020-73642-8 (PMC7538919; doi:10.1038/s41598-020-73642-8)
Supplement: Supplementary file 12 — Supplementary Information 12. [file 41598_2020_73642_MOESM12_ESM.docx]

**ASSESSMENT OF PROGNOSTIC IMPLICATION OF A PANEL OF ONCOGENES IN BLADDER CANCER AND IDENTIFICATION OF A 3-GENE SIGNATURE ASSOCIATED WITH RECURRENCE AND PROGRESSION RISK IN NON-MUSCLE-INVASIVE BLADDER CANCER_**Le Goux Constance, Vacher Sophie, Schnitzler Anne, Barry Delongchamps Nicolas, Zerbib Marc, Peyromaure Michaël, Mathilde Sibony, Yves Allory, Bieche Ivan**,** Damotte Diane, Pignot Géraldine

**Suppl. data 12:** **Immunohistochemistry results of FGFR3, RXRA and CCNE1**

| **RXRA** | **0+ (%)** | **1+ (%)** | **2+ (%)** | **3+ (%)** |
| --- | --- | --- | --- | --- |
| **All tumors (n=92)** | 9 (9.8) | 32 (34.8) | 39 (42.4) | 12 (13.0) |
| **NMIBC (n=43)** | 9 (20.9) | 14 (32.6) | 17 (39.5) | 3 (7.0) |
| **MIBC (n=49)** | 0 (0.0) | 18 (36.7) | 22 (44.9) | 9 (18.4) |

| **FGFR3** | **0+ (%)** | **1+ (%)** | **2+ (%)** | **3+ (%)** |
| --- | --- | --- | --- | --- |
| **All tumors (n=98)** | 65 (66.3) | 20 (20.4) | 10 (10.2) | 3 (3.1) |
| **NMIBC (n=45)** | 24 (53.3) | 11 (24.4) | 7 (15.6) | 3 (6.7) |
| **MIBC (n=53)** | 41 (77.3) | 9 (17.0) | 3 (5.7) | 0 (0.0) |

| **CCNE1** | **0+ (%)** | **1+ (%)** | **2+ (%)** | **3+ (%)** |
| --- | --- | --- | --- | --- |
| **All tumors (n=97)** | 2 (2.1) | 9 (9.4) | 48 (50.0) | 38 (39.6) |
| **NMIBC (n=44)** | 0 (0.0) | 4 (9.1) | 24 (54.5) | 16 (36.4) |
| **MIBC (n=53)** | 2 (3.8) | 5 (9.6) | 24 (46.2) | 22 (42.3) |
