# Supplementary material for: Assessment of prognostic implication of a panel of oncogenes in bladder cancer and identification of a 3-gene signature associated with recurrence and progression risk in non-muscle-invasive bladder cancer
Source: Sci Rep. 2020 Oct 6;10:16641. doi: 10.1038/s41598-020-73642-8 (PMC7538919; doi:10.1038/s41598-020-73642-8)
Supplement: Supplementary file 13 — Supplementary Information 13. [file 41598_2020_73642_MOESM13_ESM.docx]

**Suppl. data 13: Gene selection**

| **Gene symbol** | **Alternative symbol** | **Location** | **Accession no.** | **Gene definition** | **Function** |
| --- | --- | --- | --- | --- | --- |
| **PVRL4** | nectin-4 | 1q22-q23.1 | NM_030916 | Poliovirus receptor-related 4 | Cell adhesion |
| **MDM4** | HDMX; MDMX | 1q32 | NM_002393.4 | MDM4, p53 regulator | p53 regulation |
| **NFE2L2** | NRF2; HEBP1 | 2q31 | NM_006164.4 | Nuclear factor (erythroid-derived 2) like 2 | Antioxidant |
| **PPARG** | CIMT1; PPARG2; PPARgamma | 3p25 | NM_015869.4 | Peroxisome proliferative activated receptor, gamma | Transcription factor, cell differentiation |
| **PIK3CA** | PI3K | 3q26.3 | NM_006218.3 | Phosphoinositide 3 kinase | Cell proliferation |
| **PRKCI** | PKCI | 3q26.3 | NM_002740.5 | Protein kinase C, iota | Family of protein kinases |
| **FGFR3** | ACH; CD333; CEK2; JTK4; HSFGFR3EX | 4p16.3 | NM_000142.4 | Fibroblast growth factor receptor 3 | Fibroblast growth receptor |
| **TACC3** |  | 4p16.3 | NM_006342.2 | Transforming, acidic coiled-coil containing protein 3 | Growth and cell differentiation |
| **FBXW7** |  | 4q31.3 | NM_033632.3 | F-box and WD-40 domain protein 7 | Ubiquitination-dependent phosphorylation |
| **PAIP1** |  | 5p12 | NM_006451.4 | Poly(A) binding protein interacting protein 1 | Translational initiation |
| **TERT** | TERT; hTRT | 5p15.33 | NM_001193376.2 | Telomerase reverse transcriptase | Cellular senescence |
| **E2F3** | E2F-3 | 6p22 | NM_001949.4 | E2F transcription factor 3 | Transcription factor |
| **SOX4** | EVI16 | 6p22.2 | NM_003107.2 | SRY (sex determining region Y)-box 4 | Transcription factor |
| **EGFR** | ERBB; HER1; ERBB1 | 7p12 | NM_005228.3 | EGF receptor | Cell proliferation |
| **ZNF703** | ZNF503L | 8p11.23 | NM_025069.2 | zinc finger protein 703 | Carcinogenesis |
| **PABPC1** | PAB1; PABP; PABP1; PABPC2 | 8q22.2-q23 | NM_002568.3 | Poly(A) binding protein cytoplasmic 1 | Translational initiation |
| **YWHAZ** |  | 8q23.1 | NM_145690.2 | Tyrosine 3-monooxygenase | Transcription, transport and cell cycle regulation |
| **MYC** | c-Myc | 8q24.21 | NM_002467.4 | c-Myc oncogene | Transcription and cell cyle regulation |
| **RXRA** | NR2B1 | 9q34.3 | NM_002957.5 | Retinoid X receptor, alpha | Transcription factor |
| **GDI2** |  | 10p15 | NM_001494.3 | GDP dissociation inhibitor 2 | Regulation of family of protein kinases |
| **Ki67** | MKI67 | 10q25-qter | NM_002417 | Proliferation-related Ki-67 antigen | Cell proliferation |
| **CCND1** | BCL1 | 11q13 | NM_053056.2 | Cyclin D1 | Cell cycle |
| **HRAS** | HRAS1 | 11p15.5 | NM_005343.3 | HRAS-like suppressor | Growth factor |
| **ERBB3** |  | 12q13 | NM_001982.3 | erb-b2 receptor tyrosine kinase 3 | Receptor tyrosine kinase |
| **MDM2** | HDMX | 12q14.3-q15 | NM_002392.5 | Mdm2 protein | p53 regulation |
| **FRS2** |  | 12q15 | NM_001278351.1 | Fibroblast growth factor receptor substrate 2 | FGF receptor |
| **ERBB2** | HER2; HER-2 | 17q12 | NM_004448.3 | erb-b2 receptor tyrosine kinase 2 | Receptor tyrosine kinase |
| **CCNE1** | CCNE | 19q12 | NM_001238.3 | Cyclin E1 | Cell cycle |
| **ERCC2** | TFIIH | 19q13.3 | NM_000400.3 | ERCC excision repair 2 | DNA repair |
| **BCL2L1** | BCLX; BCL2L | 20q11.21 | NM_138578.2 | BCL2-like 1, long isoform 2 | Apoptosis |
